# New Relevant Descriptor of Linear QNAR Models for Toxicity Assessment of Silver Nanoparticles

**DOI:** 10.3390/nano10081459

**Published:** 2020-07-25

**Authors:** Alexey Kudrinskiy, Pavel Zherebin, Alexander Gusev, Olga Shapoval, Jaeho Pyee, Georgy Lisichkin, Yurii Krutyakov

**Affiliations:** 1 Department of Chemistry, Lomonosov Moscow State University, Lenin Hills 1-3, 119991 Moscow, Russia; akudrinskiy@yandex.ru (A.K.); pmzher@gmail.com (P.Z.); lisich@petrol.chem.msu.ru (G.L.); 2 National Research Center “Kurchatov Institute”, pl. Akademika Kurchatova 1, 123182 Moscow, Russia; 3 Research Institute for Environmental Science and Biotechnology, Derzhavin Tambov State University, str. Moskovskaya 10, 392000 Tambov, Russia; nanosecurity@mail.ru; 4 Department of Functional Nanosystems and High-Temperature Materials, National University of Science and Technology “MISIS”, 119991 Moscow, Russia; 5 Engineering Center, Plekhanov Russian University of Economics, Stremyanny Lane 36, 117997 Moscow, Russia; 6 Pryanishnikov Russian Scientific Research Institute of Agrochemistry, str. Pryanishnikova 31a, 127550 Moscow, Russia; shapowal.olga@yandex.ru; 7 Department of Molecular Biology, Dankook University, 119 Dandae str., Cheonan 31116, Korea; jpyee1@dankook.ac.kr

**Keywords:** silver nanoparticles, capping agent, colloidal stability, ζ-potential, toxicity, QNAR, multiple linear regression, *S. cerevisiae*, zebrafish, fungi, cyanobacteria

## Abstract

The use of silver nanoparticles (NPs) in medical, industrial and agricultural fields is becoming more widespread every year. This leads to an increasing number of experimental toxicological and microbiological studies of silver NPs aimed at establishing the risk–benefit ratio for their application. The following key parameters affecting the biological activity of silver dispersions are traditionally taken into consideration: mean diameter of NPs, surface potential of NPs and equilibrium concentration of Ag^+^. These characteristics are mainly predetermined by the chemical nature of the capping agent used for stabilization. However, the extent to which they influence the biological activity and the toxicity of silver NPs varies greatly. In this work, dispersions of silver NPs stabilized with a wide array of substances of different chemical nature were used for quantitative evaluation of whether the various measurable properties of silver NPs fit as descriptors of linear QNAR (quantitative nanostructure–activity relationship) models for silver NP toxicity evaluation with respect to a model eukaryotic microorganism—*Saccharomyces cerevisiae* yeast cells. It was shown that among the factors that determine silver NP toxicity, the charge of particles, their colloidal stability and the ability to generate Ag^+^ ions carry more importance than the descriptors related to the particle size. A significant synergistic effect between the ζ-potential and the colloidal stability of silver NPs on their toxicity was also discovered. Following this, a new descriptor has been proposed for the integral characterization of the silver dispersion colloidal stability. According to the obtained data, it can be considered applicable for building QNAR models of higher efficacy. The validity testing of the proposed model for theoretical prediction of silver NP toxicity using a wide range of living organisms has shown that this new descriptor correlates with toxicity much better compared to most traditionally used descriptors. Consequently, it seems promising in terms of being used not only in situations involving the rather narrow array of the objects tested, but also for the construction of silver NP toxicity models with respect to other living organisms.

## 1. Introduction

Metal nanoparticles (NPs), especially silver NPs, are widely used in production of various household and industrial goods [1,2,3]. Currently, the range of application of silver NPs in the field of human and veterinary medicine is gradually expanding, leading to their use as bacteriostatic, anti-inflammatory and wound-healing components for numerous medical devices [4,5,6], as well as plant protection products in agriculture [7,8,9,10]. Wide-scale use of any type of NPs is associated with the risk of their release into and accumulation in the environment [11,12]. This in turn requires the development and improvement of procedures for adequate evaluation of potential risks associated with an environmental exposure to NPs [13].

Using experimental toxicological and microbiological methods to determine effectiveness and safety of newly synthesized nanoparticles can be expensive and time-consuming, since they require a variety of biological tests to evaluate their effects on a wide range of types of microorganisms, plants, aquatic and soil organisms, insects and endothermic animals [14,15,16,17,18]. Scientific experience has shown that the general results of many experiments can often be predicted based on certain parameters of nanomaterials (mean diameter of NPs, absolute value and sign of zeta-potential of NPs, and equilibrium concentration of Ag^+^) [19,20]. In addition to saving resources and time, this also helps in solving a serious ethical problem—the unjustified use of laboratory animals in such studies [21]. In this case, various empiric models can be of help instead to predict the toxicological and microbiological activity of NPs based on their structure and properties, i.e., QNAR models (quantitative nanostructure–activity relationship). Similar with QSAR models (quantitative structure–activity relationship), which are used for organic compound activity prediction, the descriptors typically used to make linear QNAR models as well as models based on neural net methodology characterize the nanoobject structure: the chemical composition of a nanoparticle core, the capping agent structure and similar characteristics [19,20,22,23,24,25,26].

Nevertheless, the practice shows that NPs are extremely complex objects, and their properties are not always fully predictable even with their component characteristics known. From our point of view, relevant QNAR models necessarily utilize not only the traditional descriptors characterizing nanoobject component properties, but also the descriptors characterizing the entire object properties. Such integral descriptors include, for example, the electrical charge of the NP as a whole, meaning both the core and the stabilizing layer charges, as well as the charge of the closest counter-ion layer in the solution [27,28]. Among other integral descriptors, there are parameters of interaction between NPs and the solution that contains them, namely the colloidal or aggregate stability in the presence of some coagulants and organic molecules, which are inevitably present during trials. Furthermore, such kinetic parameters of NP behavior in a solution include the speed of NP dissolution or rates of other chemical processes involving the dispersion medium components [29,30].

Due to this, the search for the significance evaluation and the selection of descriptors maximally correlating with the biological activity of NPs becomes one of the most important stages of making relevant QNAR models. For the purpose of experimental justification of such models, it is necessary to use different in vitro methods of assessment of nanomaterials [31]. Among these methods is the evaluation of activity against prokaryotic cells—Gram-positive and Gram-negative bacteria [32], eukaryotic cells—microalgae [33], fungi [34], yeast [35], and especially various animal and human cell lines [28]. In our experiment, we chose *Saccharomyces cerevisiae* cells for several reasons. *S. cerevisiae* is one of the most commonly used model simplest eukaryotic organisms [36], especially in the field of silver NP research [37,38,39]. The use of *S. cerevisiae* as the model organism is also an important distinguishing feature of most evidence-based biological studies [40], because it allows one to: a. overcome existing ethical and experimental barriers that inevitably arise while working with certain target organisms (e.g., mammals); b. apply the results obtained during a model experiment to a wide range of organisms of various classes among which the studied biological process is common [41].

In this study, eukaryotic *S. cerevisiae* cells and the aqueous dispersions of silver NPs stabilized with an uncharacteristically wide array of positively and negatively charged surfactants and polymers as well as their uncharged counterparts were used for quantitative evaluation of whether the various experimentally measured properties of silver NPs are suitable as QNAR (quantitative nanostructure–activity relationship) linear model descriptors for toxicity assessment of silver NPs. We also conducted further assessment of the theoretical significance of various descriptors using experimental data related to the toxicity of silver NPs with respect to zebrafish embryos (*Danio rerio,* freshwater fish belonging to the Cyprinidae family), mycelial phytopathogenic fungi (*Alternaria solani* and *Rhizoctonia solani*) and freshwater cyanobacteria *Synechocystis* sp. PCC 6803.

## 2. Materials and Methods

Glucose (99%, Acros Organics, Morris Plains, NJ, USA), potassium chloride (99%, Acros Organics, USA), sodium hydroxide (99%, Sigma-Aldrich, St. Louis, MO, USA), ammonia (25% aqueous solution, Sigma-Aldrich), ethoxylated polydimethylsiloxane (99%, Wacker Chemie AG, Nuenchritz, Germany), polydimethyldiallylammonium chloride (20% aqueous solution, Sigma-Aldrich, USA), nonylphenol ethoxylated (99%, Parchem, New Rochelle, NY, USA), coco trimethylammonium methoxysulfate (50% aqueous solution, Kao Corporation S.A., Barcelona, Spain), polyoxyethylenesorbitan monooleate “Tween-80” (99%, Gee Lawson Chemicals, Cheshire, UK), benzyldimethyl [3-(myristoylamino)-propyl] ammonium chloride monohydrate (99+%, PharmChem, Fort Worth, TX, USA), xanthan gum (99%, Deosen Biochemical Ltd., Ordos, China), polyhexamethylene guanidine hydrochloride (99%, Parchem), polyhexamethylene biguanide hydrochloride (20% aqueous solution, Arch Chemicals Inc., Castleford, UK), poly(acrylic acid) (99%, Acros Organics), sodium α-olefin sulfonate (97%, TNJC, Taiwan, China), sodium dodecyl sulfate (99%, Sigma-Aldrich), polyoxyethylated aliphatic alcohol C_12_–C_14_ (97%, Mistral Industrial Chemicals, Antrim, UK), sodium coco dipropionate (38% aqueous solution, Lakeland Laboratories Ltd., Manchester, UK), sodium tallow amphopolycarboxyglycinate (30% aqueous solution containing 10% NaCl, Akzo Nobel, Sundsvall, Sweden), didecyl dimethylammonium chloride (80% solution in 2-propanol, Akzo Nobel), sodium dodecyl sulfoethoxylate (70% aqueous solution, Hansa, Nuremberg, Germany), sodium borohydride (99%, Acros Organics), silver nitrate (99+%, Ural Chemical Reagents Plant, Verkhnyaya Pyshma, Russia), trisodium citrate dihydrate (99%, Sigma-Aldrich), and hydrogen peroxide (30% aqueous solution, Sigma-Aldrich) were used as received. Doubly distilled water was used for the preparation of all solutions during each trial.

### 2.1. Synthesis of Silver NPs Stabilized with Different Capping Agents

Most of the aqueous nanosilver dispersions were obtained by the common method of AgNO_3_ reduction using sodium borohydride in the presence of a stabilizer [42]. The capping agents of different chemical nature used for stabilization of silver NPs are listed in Table 1.

A minimal amount of the stabilizer needed to prepare a dispersion of nanosilver with high colloidal stability and without a tendency for NP aggregation within a period of the first three months was added to the reaction mixture (Table 1).

### 2.2. Preparation of Aqueous Dispersions of Benzyldimethyl [3-(Myristoylamino)-Propyl] Ammonium Chloride-Stabilized Colloidal Silver by the Glucose Reduction Method

To 50 mL of 0.017 g/L (0.1 mM) silver nitrate aqueous solution, 150 µL of 0.01% (38 mM) sodium hydroxide was added [43]. The resulting silver (I) oxide precipitation was dissolved by adding 50 µL of 25% ammonia solution.

2AgNO_3_ + 2NaOH = 2NaNO_3_ + Ag_2_O + H_2_O (1)

Ag_2_O + 8NH_3_ + H_2_O = 2[Ag(NH_3_)_4_]OH (2)

An amount of 50 mL of an aqueous solution containing 0.010 g (0.02 mmol) of benzyldimethyl [3-(myristoilamino)propyl] ammonium chloride was added dropwise (with vigorous mixing) to the obtained solution. 15 min after adding the stabilizing solution, 0.35 g (1.9 mmol) of glucose was added into the reaction mix, which was then heated for 30 min to 49 °C.

2[Ag(NH_3_)_4_]OH + C_6_H_12_O_6_ = 2Ag + 3NH_3_ + C_5_H_11_O_6_COONH_4_ + H_2_O (3)

All obtained dispersions containing residues of the reducing agent and the products of its oxidation were purified using a dialysis technique. Each procedure involved the immersion of a dialysis bag (Servapor^®^, pore diameter 2.5 nm, SERVA Electrophoresis GmbH, Heidelberg, Germany) containing 100 mL of a dispersion of silver NPs in a glass beaker containing 1 L of an aqueous solution of a capping agent with the target concentration of *c (St)* (see Table 1) and being stirred with a magnetic stirrer for 24 h without access to air. The dialysis procedure was repeated twice in identical conditions, increasing the impurities reduction degree to 10^2^. Purified aqueous dispersions of silver with the concentration of 100 μg/mL were stored in the dark at room temperature in hermetically sealed plastic containers without access to air.

### 2.3. Transmission Electron Microscopy (TEM)

Electronic images and microdiffraction patterns were obtained using a Leo 912 AB Omega transmission electron microscope (LEO Electron Microscopy Inc., NY, USA) with the accelerating voltage of 100 kV in accordance with the standard procedure described in [42], as well as a Carl Zeiss NVision 40 scanning electron microscope (Carl Zeiss Group, Oberkochen, Germany) equipped with an X-Max detector (Oxford Instruments, Abingdon, UK) with the accelerating voltage of 7 kV. Samples were prepared by applying 1–2 μL of the dispersion on a carbon grid, which was then air-dried.

### 2.4. Dynamic Light Scattering (DLS)

The ζ-potential of the obtained silver NP dispersions was determined using the time-resolved dynamic light scattering (DLS) technique involving the Photocor Complex (Photocor LLC, Moscow, Russia). In each case, dispersions of silver NPs (2 mL) with an Ag concentration of 15 μg/mL were studied. All measurements were carried out at a scattering angle of 90 degrees. Each autocorrelation function was accumulated over 15 s, and the intensity weighted hydrodynamic size was then obtained using the second-order cumulant analysis method (DynaLS v.2.0 software, Alango, Israel). This particular method helped to calculate diffusion coefficients of silver NPs in accordance with the correlation function approximation algorithms, based on the assumption that NPs have monomodal Gaussian size distribution [44].

### 2.5. X-ray Phase Analysis (XRD)

X-ray images were obtained by examining the thoroughly washed and dried silver NP powder prepared by centrifuging an aqueous colloidal solution using a Bruker D8 Advance X-ray diffractometer (Bruker Corp., Billerica, MA, USA) (in the Bragg-Brentano geometry) with the radiation from the Cu_Kα_ anode. Diffraction peaks were identified using the JCPDS database (International Centre for Diffraction Data, USA). The calculation of the sizes of coherent scattering regions (CSRs) of nanocrystalline silver samples was carried out according to the Scherrer formula:

(4) $$D_{hkl}=\frac{K\cdot \lambda }{\left[\beta_{hkl}2\theta -s\right]\cos\theta }$$

where *θ* is the position of the peak maximum, *λ* is the X-ray wavelength of Cu_Kα_ (0.154056 nm), *β_hkl_* (2*θ*) is the total physical broadening of the diffraction maximum, and *s* is the instrumental broadening (0.1°). The value of the Scherrer constant (*K*) was considered equal to 1. To determine the value of *β* after the background subtraction, the calculation of the experimental X-ray peak (111) of silver using the pseudo-Voigt function was conducted.

### 2.6. Spectrophotometric Measurements

Spectrophotometric measurements in the range of 300–700 nm wavelengths were performed on a UV-1800 spectrophotometer (Shimadzu Corp., Kyoto, Japan) using quartz cuvettes with a path length of 10 mm.

### 2.7. Evaluation of the Colloidal Stability of Nanosilver Aqueous Dispersions in the Presence of KCl

25 µL of 3 M solution of potassium chloride was added using a pipet pump and mixed into 50 mL of a dispersion containing 50 mg/L of silver. The optical absorption spectrum of the mix was recorded. KCl solution was added before the start of the dispersion coagulation. Reduction in the intensity of the characteristic absorption band of silver NPs by more than 5% at 440–460 nm for the dispersion stabilized by polyacrylic acid and at 400–420 nm for other dispersions was considered to be the sign of the onset of coagulation. Nevertheless, the decrease in the intensity of the characteristic absorption band was no more than 3–4% for all dispersions within 3 months from the moment of synthesis. Thus, the obtained dispersions can be considered colloidally stable during a long period of time.

### 2.8. Microbiological Experiments

#### 2.8.1. Experimental Evaluation of the Yeast Growth Suppression

*S. cerevisiae* cells (strain VKM Y-1173) were obtained from the collection of the Institute of Physiology and Biochemistry of Microorganisms (RAS, Pushchino, Russia). The cells were cultivated in a YNB synthetic medium (Millipore^®^, Burlington, MA, USA) placed on a shaker at 29 °C until the middle of the log phase was reached [45]. After that, the yeast cells were washed twice with distilled water. Before the start of the experiment, a suspension of the cells with the optical density of *D_600_* = 6.0 was diluted 50 times.

A sterile pyrogen-free SPL 96 well plate (SPL Life Sciences Co., Ltd., Gyeonggi-do, Korea) was used to evaluate the minimum inhibitory concentration (MIC) of silver NP dispersions. A 40 μL dispersion of NPs and a 10 μL suspension of the yeast culture were introduced into the wells in triplicate. Incubation process lasted 1 h at room temperature. Then 250 μL of the YNB medium, additionally containing 2% glucose, was introduced into each well. The first reference experimental run was triplicated and involved wells containing *S. cerevisiae* cells in a nutrient medium without NPs. One empty well was used to set the baseline when measuring the optical density. The second reference experimental run involved three wells without the cells. The well plate was kept in the vibration mode for 24 h at 37 °C, then the second reference experimental run took place: the yeast culture was introduced into the nutrient medium immediately before recording the absorption spectra of the cell culture suspensions. The optical density of *λ* = 600 nm was measured using a Multiskan Sky microplate spectrophotometer (Thermo Fisher Scientific Inc., Waltham, MA, USA). The suppression of the yeast growth was confirmed by a decrease in the optical density compared to the reference run cells, which was registered when the silver MIC was reached.

#### 2.8.2. Experimental Evaluation of the Mycelial Fungi Growth Suppression

*Alternaria solani* Sorauer and *Rhizoctonia solani* J.G. Kühn pure cultures were obtained from the collection of the Faculty of Biology, Lomonosov Moscow State University, Moscow, Russia. To evaluate the fungistatic effect of silver NP dispersions, an experimental technique previously described in [46] was used. Cultures of the fungal strains were sectioned into small agar inoculum blocks (5 mm in diameter) that were placed in the center of Petri dishes containing 25 mL of malt extract agar (Sigma-Aldrich) with 0.1; 1; 10; and 100 µg/mL of silver NPs added. Malt extract agar without silver NPs was used during the reference experimental run. Inoculated dishes were incubated at 24 ± 1 °C in the dark and the colony diameters were measured (in two directions in the case of each dish) after 18–25 days of growing, when the referential colony had a diameter of approximately 50 mm. The mycelial growth rate of each isolate was used to calculate the inhibition percentage as compared with that of the referential colony. The silver NP concentration providing the inhibition rate of 50% more than what was observed during the reference run (EC_50_) was determined for both isolates using the method of linear interpolation. Each experiment was performed in triplicate.

#### 2.8.3. Assessment of the Silver NP Activity against Cyanobacteria

The wild type of *Synechocystis* sp. PCC-6803 pure culture was obtained from the collection of the Faculty of Biology, Lomonosov Moscow State University, Moscow, Russia. Bacterial cells were cultivated at 30 °C in a BG-11 synthetic medium (Sigma-Aldrich) containing NaNO_3_ 15 g/L, K_2_HPO_4_ 0.4 g/L, MgSO_4_·7H_2_O 0.75 g/L, CaCl_2_·2H_2_O 0.36 g/L, citric acid 60 mg/L, ammonium iron citrate 60 mg/L, EDTA 10 mg/L, Na_2_CO_3_ 0.2 g/L, H_3_CO_3_ 28.6 mg/L, MnCl_2_·4H_2_O 18.1 mg/L, ZnSO_4_·7H_2_O 2.22 mg/L; Na_2_MoO_4_·2H_2_O 3.9 mg/L, CuSO_4_·5H_2_O 0,79 mg/L, Co(NO_3_)_2_·6H_2_O 4.94 mg/L aqueous solution [47]. The *Synechocystis* sp. PCC-6803 cells were cultivated for 4 days under continuous white fluorescent light at 40 µmol photons per m^2^ × s [48] in the BG-11 broth medium with the silver NP dispersion added (or without it in the case of the reference run). The optical density of the cell culture was measured at room temperature and with *λ* = 540 nm using a Cary-Bio 300 spectrophotometer (Varian Medical Systems, Inc. Palo Alto, GA, USA). The suppression of the bacterial growth was confirmed by a decrease in the optical density compared to the reference run cells, which was registered when the silver MIC was reached. Each experiment was performed in triplicate.

## 3. Results and Discussion

### 3.1. Synthesis of Dispersions of Silver Nanoparticles

Chemical reduction in ionic silver with sodium borohydride is one of the most popular methods of obtaining silver NPs in both homogeneous and heterogeneous systems [49]. Sodium borohydride, unlike other reducing agents (including citric and ascorbic acids, polyphenols, simple carbohydrates, hydrazine hydrate, hydroxylamine hydrochloride, etc.), has high reactivity, which allows the reduction process to be carried out quickly and under total control. The flexibility of the borohydride reduction method made it possible for the silver NP synthesis technique to be improved using capping agents of different chemical classes, including (see Table 1) anionic, amphoteric, nonionic, cationic surfactants and positively or negatively charged polymers [10,50,51].

**Figure 1:**
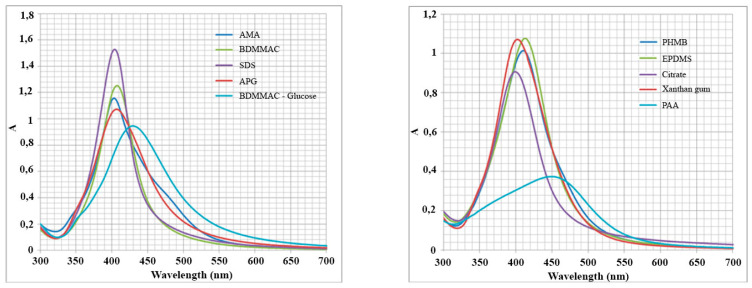
Differences in the absorption spectra of the dispersions of silver NPs; A is the optical density.

Figure 1 shows the UV-vis absorption spectra of most of the prepared colloids. The position of the characteristic bands suggests a non-covalent (mostly electrostatic and coordinative) interaction of the stabilizers with the surface of silver NPs [52].

The concentration of the stabilizer in each dispersion is shown in Table 1. All obtained dispersions had high colloidal stability. The intensity of the silver NP absorption band remaining at 95% of the original value or more over the period of no less than 3 months was chosen as the criteria for the dispersion’s ability to resist aggregation.

**Figure 2:**
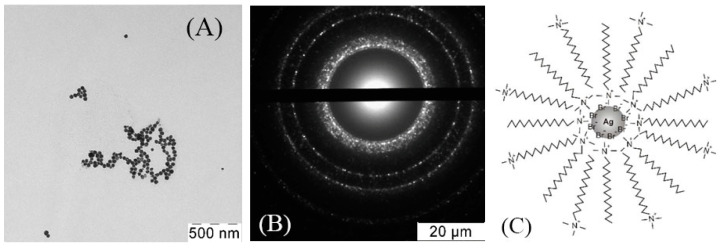

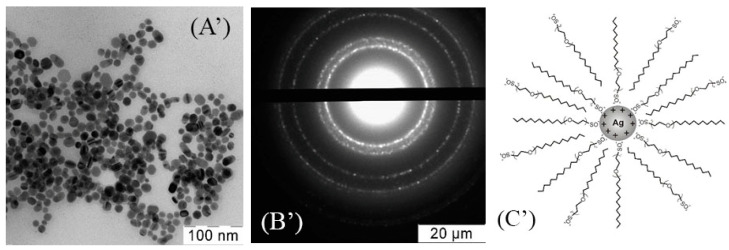
(**A**) The typical TEM images, (**B**) electron microdiffraction patterns, and (**C**) schematic representation of the stabilizing layer of the BDMMAC with glucose as the reducing agent and SLES-stabilized silver NPs (**A’**), (**B’**), (**C’**).

Data on size distribution of silver NPs in the dispersions estimated from the TEM images appear to align with the results of the dynamic light scattering measurements. Figure 2 shows the data on silver particles stabilized by sodium laureth sulfate (SLES) and benzyldimethyl [3-(myristoylamino)-propyl] ammonium chloride (BDMMAC).

Typical electron microdiffraction patterns for all NPs (Figure 2) indicate that NPs are composed of the crystalline silver phase.

**Figure 3:**
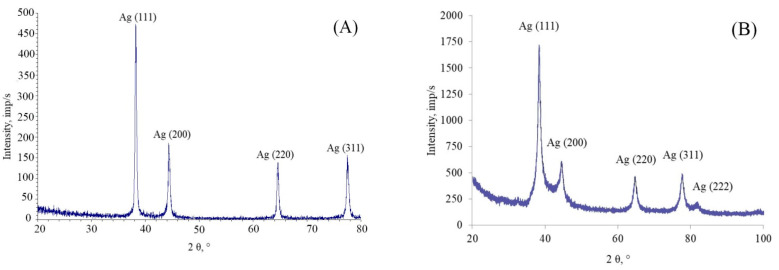
Typical X-ray diffractograms of the irreversibly coagulated silver NPs stabilized with (**A**) BDMMAC (with glucose as the reducing agent), and (**B**) PHMB, with the characteristic peaks corresponding to interplanar distances of the crystalline silver.

X-ray diffractogram shows only the peaks being similar to those of the crystalline silver (Figure 3 shows the diffractogram for the SLES- and BDMMAC-stabilized silver particles only; the diffractograms for other particles are similar).

### 3.2. Descriptor Selection for the QNAR Model Building

Biological properties of silver NPs directly depend on their size [25,53,54]. Thus, the mean size of particles in the dispersion has been chosen as the first potential descriptor.

The diameters of silver NPs in the dispersions under study estimated from the analysis of the TEM images are summarized in Table 2.

The majority of resulting colloidal solutions of silver, excluding dispersions stabilized with polyacrylic acid (PAA, large particles) and polyhexamethylene biguanide hydrochloride (PHMB, small particles) have quite a narrow range of particle size distribution and the maximum percentage of particles have the diameter of 7–11 nm.

The size distribution of particles can also have a significant impact on the action of colloidal silver. In particular, this may be due to the different efficacy of cell membrane binding for particles of various sizes. Moreover, the biological action of silver NPs, as shown in our previous work [55], depends on the NP dissolution speed followed by the release of much more active silver ions. Conversely, the dissolution speed is different for large and small particles and it depends on the particle surface area. With this considered, a specific area of the dispersion particle surface has been chosen as an additional descriptor. This parameter combined with the mean particle size is a heterogeneity marker of silver NPs.

Specific surface area of silver NPs in the dispersion *S*_sp_ was calculated according to [55] and summarized in Table 2. The mean surface *s* and the mean volume *w* of the particles in the dispersion were calculated from the size distribution of the dispersion through the following equations $s=\underset{i}{\sum }\varphi_{i}\pi d_{i}^{2}$, where *φ_i_* is the fraction of particles with diameter *d_i_* in the dispersion according to the size distribution histograms; $\pi d_{i}^{2}$ is the surface area of a particle with diameter *d_i_*; and $w=\underset{i}{\sum }\varphi_{i}\frac{1}{6}\pi d_{i}^{3}$, where $\frac{1}{6}\pi d_{i}^{3}$ is the volume of a particle with diameter *d_i_*; *S*_sp_ (m^2^/g) was calculated through the equation $S_{sp}=\frac{s}{w\rho }=\frac{6\sum_{i}\varphi_{i}d_{i}^{2}}{\rho \sum_{i}\varphi_{i}d_{i}^{3}}$, where *ρ* is the density of silver.

The activity of silver NPs has a significant correlation with the particle charge as the interaction between silver NPs and charged cell walls plays an important role in the biological action of dispersions [25,56,57]. The value of electrokinetic ζ-potential can serve as the quantitative description of the charge of particles, which is why it was chosen as another potential descriptor.

The experimental values of the ζ-potential of silver NPs for all aqueous dispersions were calculated using the DLS technique and are presented in Table 2. Measurement of the ζ-potential of silver NPs is widely used to assess the colloidal stability of dispersions [58,59]. The ζ-potential of 30 mV (either positive or negative) can be used to distinguish “low-charged” surfaces from “high-charged” surfaces at the electrokinetic slipping plane being the hypothetical border between mobile and immobile parts of the diffuse layer. The higher the absolute value of the electrokinetic potential, the more stable the colloid system is supposed to be. The electrokinetic potential of silver NPs depends on the chemical structure of the capping agent both in terms of its absolute value and its sign (see Table 1). Thus, in the case of nonionic surfactants (both of big and small molecular weight) the absolute value of the ζ-potential is low while its sign can vary. Surface modification of NPs with cationic surfactants and positively charged polymers leads to positive values of the ζ-potential, while the anionic surfactants produce negative values; amphoteric surfactants in a slightly alkaline medium (pH = 7.5–8.5) produce the negative ζ-potential of the highest absolute value. Indeed, the experimentally calculated ζ-potential of sodium coco aminodipropionate (AMA)-stabilized silver NPs had the highest absolute value equal to –56 ± 1 mV; NPs stabilized with nonionic ethoxylated polydimethylsiloxane (EPDMS) had the lowest absolute value of +8 ± 1 (see Table 2).

The value of ζ-potential can only be used to describe the colloidal stability of recently prepared dispersions. Upon interacting with biological objects, silver NPs are surrounded by several substances that can potentially cause coagulation and subsequent inactivation of nanosilver [60,61]—for example, a protein corona. Therefore, correct evaluation of biological action (e.g., toxicity) requires descriptors that characterize the dispersion stability directly in biological media.

It is well known that electrolytes, especially ions adsorbed on the particle surface, significantly affect the colloidal stability of silver NPs [62]. Such ions, in particular, include chlorides and phosphates present both in biological fluids, underground water and water basins. Therefore, *c_max_* (KCl), the maximum KCl concentration in the dispersion when no significant NP coagulation has yet been observed, was chosen as an easily measured parameter for the nanosilver dispersion resistance to electrolyte effects. The measured values of *c_max_* (KCl) are shown in Table 2. Dispersions obtained in this study were significantly different in their resistance to the KCl effects. The lowest resistance *c_max_* (KCl) of <40 mM was registered for silver NPs stabilized with sodium citrate and with anionic surfactants (SLES and SDS). Apparently, Cl^−^ ions can effectively force other anions out of the silver surface, thus destroying the stabilizing layer.

Colloidal stability of silver NPs capped with cationic surfactants and ammonium chloride-based polymers used in this study is due to the layer of bulky positive ions, while the silver particle surface itself becomes covered with chloride ions at the stage of NP formation. This increases their resistance to KCl, *c_max_* (KCl) being 40–300 mM. Nevertheless, the additional adsorption of chloride anions on the surface of positively charged NPs reduced their total charge, thus leading to particle aggregation.

The highest resistance to KCl (*c_max_* (KCl) ≥ 480 mM) can be observed in the case of dispersions of negatively charged NPs stabilized with amphoteric carboxyalkylamine-based surfactants: AMA and sodium tallow amphopolycarboxyglycinate (STAPCG). This is due to the high affinity of polyamines to the silver surface making it difficult for chloride ions to force surfactant ions out of the silver surface.

A variety of studies demonstrate that Ag^+^ ions formed in an oxidative dissolution of silver NPs play an important role in the biological activity of silver NP dispersions. For several obtained dispersions there are quantitative literature data on the rate of silver NP oxidation with hydrogen peroxide, an oxidizer that is being constantly formed and exists in living cells [55,63]. These data could also be used as a descriptor for QNAR model building. The data from our recent study [55] are also shown in Table 2.

Table 2 contains experimental data characterizing structural, electrochemical and kinetic parameters of the dispersions obtained. These data characterize nanoobjects as a whole, including the stabilizing layer, and they can be used as potential descriptors for QNAR models describing the biological action of silver NPs. Furthermore, an analysis of the influence of these parameters on the dispersion toxicity with respect to yeasts will determine how relevant these descriptors are in predicting the biological activity of silver NPs.

The silver NP colloids were also screen-tested with regard to their MICs towards *S. cerevisiae* (VKM Y-1173 strain). Minimum inhibitory concentration (MIC) is the lowest concentration of an antimicrobial substance which causes inhibition of the growth of colonies of microorganisms after 24 h of incubation in a growth medium. MIC can either be seen with the naked eye or determined spectrophotometrically [64]. MICs of silver NPs are most often used as a convenient research tool to evaluate the in vitro activity against bacteria and yeasts.

From the early 2000s with the development of the experimental methods in molecular biology, the number of research tools using *S. cerevisiae* as a model biological object has increased significantly. This was mainly due to the accumulated fundamental knowledge of the physiology, biochemistry, the structure of the cell wall and the cytoskeleton, genome sequencing and proteome profiling of these simplest eukaryotic organisms [40]. *S. cerevisiae* is also a convenient model organism for cytotoxicity assessment because, in contrast to more complex eukaryotes, the cells of this yeast can be easily cultivated in simple growth media, keeping the parameters of local environment and physicochemical conditions constant. Up to this day there has been a large number of scientific studies aimed at the assessment of the activity of NPs in relation to prokaryotic organisms; however, the majority of them involve commonly spread microbiological tests, which leaves little room for further understanding of the mechanisms of action of silver NPs towards living cells. At the same time, the number of studies on eukaryotic cell lines remains moderate, and the results of many of them are rather contradictory [65,66]. The inconsistency of some results, in our opinion, is due to the fact that the authors do not take into account the key colloidal characteristics of the materials under study as well as their behavior in the local environment (e.g., a culture medium) when planning a biological experiment. The combination of these facts prompted us to assess the biological activity of silver NPs using eukaryotic *S. cerevisiae* cells.

The data on the biological activity (MIC) are summarized in Table 3. The MIC value of each stabilizer used significantly exceeded the MIC value of the respective dispersion of silver NPs capped with the respective stabilizer, which shows that the studied dispersion toxicity towards *S. cerevisiae* is due to the activity of silver NPs.

### 3.3. Evaluation of Applicability of Different Silver NP Characteristics as Descriptors for Building QNAR Models

Adequate selection of descriptors is a necessary step in building a high-quality QNAR model. Descriptors should have a significant impact on the parameters predicted with the model; their values should be significantly different for nanostructures in the range selected for model building. In addition, the descriptors should not correlate with each other.

For testing the effects of potential descriptors listed in Table 2 on a target parameter (toxicity to yeast cells), Pearson linear correlation coefficients (*r*) between descriptors and minimum inhibiting concentration logarithms (log_10_ (MIC)) from Table 3 have been calculated. For testing the possible descriptor intercorrelation, Pearson linear correlation coefficients (*r*) between each pair of descriptors from Table 2 have been calculated. The results are shown in Table 4.

Table 4 shows the lack of correlation between the selected descriptors (|*r*| < 0.5), excluding the pair of *d*-*S_sp_.* Therefore, building the relevant QNAR model requires excluding one of the descriptors from this pair. Table 2 demonstrates stronger correlation between the particle size *d* and the target parameter, i.e., toxicity to yeast cells, the value of correlation coefficient in the *d*-log_10_ (MIC) pair being −0.11 and in the *S_sp_*-log_10_ (MIC) pair being −0.06. Therefore, selecting the particle size *d* as a descriptor would be more appropriate. Even though the correlation between biological activity and the size of silver NPs is well-noted in the literature, it should be mentioned that according to the data obtained in this study, the particle size and specific surface are the least significant descriptors with regards to the biological activity.

At the same time, the maximum correlation *r* = −0.41 has been demonstrated between the NP toxicity to yeast cells, log_10_ (MIC), and the hydrogen peroxide oxidation rate constant *k*‘ of silver NPs, hydrogen peroxide being the biologically significant oxidizer. It correlates well with the findings of the studies [55,67] on the biological activity of silver NP dispersions being dependent on the Ag^+^ ion concentration and on the rate of Ag^+^ ion generation during the oxidative dissolution of NPs.

The average values of the descriptor influence on the target parameter of log_10_ (MIC), |*r*| < 0.3–0.5, are observed in the case of descriptors characterizing the aggregate stability and the electrochemical properties of NPs: *c_max_* (KCl) and the ζ-potential. Nevertheless, cases of higher influence of the particle charge on the biological activity are widely reported in the literature. This is due to both the electrostatic attraction and the efficacy of silver particles binding to the mostly negatively charged yeast and bacterial cell surface being mostly determined by the NP charge. It is the positively charged particles stabilized with didecyl dimethylammonium chloride (DDDMAC) and benzyldimethyl [3-(myristoylamino)-propyl] ammonium chloride (BDMMAC) that have the lowest MIC value of 3.12 µg/mL, thus demonstrating the highest toxicity to yeast cells.

**Figure 4:**
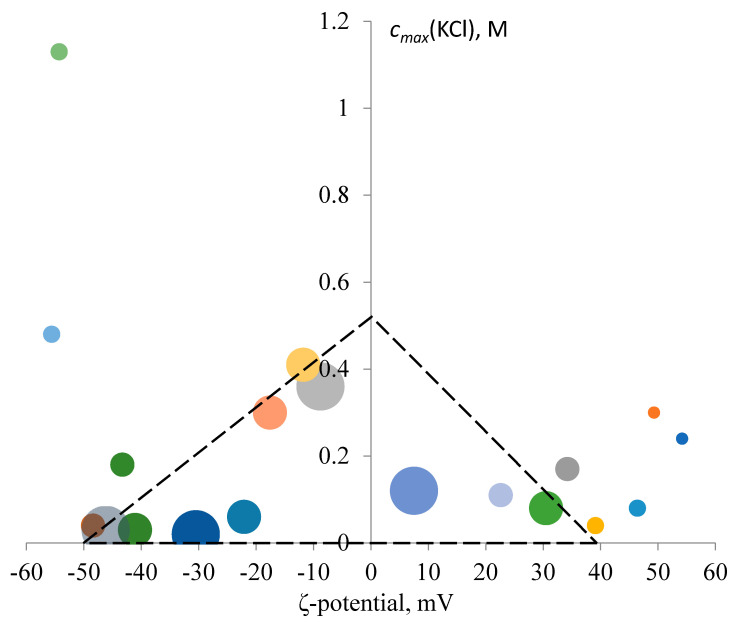
Effects of the ζ-potential and the silver NP resistance to KCl, *c_max_* (KCl), on the NP toxicity to yeast cells. The size of each bubble is proportional to the MIC value of the corresponding dispersion; the smaller the size, the higher the biological activity of the dispersion.

The relation between *c_max_* (KCl), the ζ-potential and biological activity needs to be examined in more detail. Figure 4 shows a bubble chart (ζ and *c_max_* (KCl) as the *x* and *y* axes respectively) where points characterize the dispersions obtained.

Figure 4 clearly shows that despite the lack of significant correlation between *c_max_* (KCl), the ζ-potential and log_10_ (MIC), the dispersion values are not uniformly spread along the diagram. The NPs with high values of biological activity (little bubbles) are mostly located in the areas corresponding both to the high ζ-potential and the high resistance to KCl. On the other hand, NPs with low values of biological activity are mostly located along the *c_max_* (KCl) = 0 axis or the ζ = 0 axis.

Thus, it can be concluded that the significant biological activity of silver NPs equally requires the specific (either negative or positive) electrical charge of about |ζ| > 10 mV and a certain level of resistance to electrolytes, i.e., *c_max_* (KCl) > 120 mM.

Generally, NPs with high cytotoxicity (MIC < 12.5 µg/mL) are located beyond the so-called “magic” triangle area with the corners corresponding to the following values: ζ = +40 mV, ζ = –60 mV and *c_max_* (KCl) = 500 mM.

These observations confirm the direct connection between the particle charge, the dispersion resistance to electrolytes and the biological activity of NPs. However, the selected set of descriptors is insufficient for fully taking this connection into account, since it contains no parameters characterizing the combined effect of the ζ-potential and *c_max_* (KCl). This explains quite low correlation coefficients in the *c_max_* (KCl)-log_10_ (MIC) and ζ-log_10_ (MIC) pairs.

To surpass this obvious scarcity of the descriptor set and to allow the adequate prediction of biological activity within QNAR linear models, we suggest including a new descriptor that characterizes the combined effect of the particle charge and the dispersion resistance to electrolytes. Out of all possible descriptors, we suggest the product ζ × *c_max_* (KCl) or its absolute value |ζ| × *c_max_* (KCl). The values of ζ × *c_max_* (KCl) and |ζ| × *c_max_* (KCl) are low along the axes of *c_max_* (KCl) = 0 and ζ = 0, while the highest values of these new descriptors correspond to the dispersions characterized both by high electrolyte resistance and by a high electrical charge.

In the pairs ζ × *c_max_* (KCl)-log_10_ (MIC) and |ζ| × *c_max_* (KCl)-log_10_ (MIC), the values of correlation coefficients are 0.07 and -0.48 respectively; this means that the |ζ| × *c_max_* (KCl) descriptor correlates with the biological activity better than the ζ-potential or *c_max_* (KCl) separately. Such a significant difference between ζ × *c_max_* (KCl)-log_10_ (MIC) and |ζ| × *c_max_* (KCl)-log_10_ (MIC) descriptors is due to the significant biological activity correlating not with the electric charge sign of the NP (as it was mentioned before), but rather with the presence of a sufficient positive or negative charge providing the minimum aggregate stability of the dispersions, i.e., |ζ| > 10 mV.

Before using the new descriptor, |ζ| × *c_max_* (KCl), in a QNAR model, one should check the lack of its significant correlation with other descriptors. Table 4 shows the mutual correlation in the |ζ| × *c_max_* (KCl)-*c_max_* (KCl) pair only, *r* = 0.93, which makes the use of the *c_max_* (KCl) descriptor unnecessary.

### 3.4. Building of Linear QNAR Models Based on Proposed Descriptors

A set of 19 obtained and experimentally characterized dispersions is insufficient for building a fully-fledged QNAR model and its validation with training and testing sets. However, it is perfectly valid for demonstration of the possibility of building such model based on the selected descriptors and for the evaluation of descriptor relevance.

In QNAR studies, the most desirable model is simple, direct, reliable, and easy to explain. To gather that, one usually resorts to the multiple linear regression (MLR) method when there are two or more than two independent descriptors.

We built some MLR models deriving a mathematical function which best describes the desired biological activity as a linear combination of the descriptors with the regression coefficients.

(5) $$log_{10}\left(MIC\right)=a_{0}+\sum_{i}a_{i}p_{i}$$

where *p_i_* is the value of *i*-th descriptor, *a_i_* is the corresponding regression coefficient, and *a_0_* is the residual coefficient. We computed the regression coefficients using the ordinary least squares method. This procedure was implemented using the Origin 2016 software (OriginLab Corp., Northampton, MA, USA).

The results of QNAR linear model building based on the selected descriptors and the statistical characteristics of model accuracy are shown in Table 5.

Table 5 shows that QNAR models No. 3 and 4 based on the suggested new descriptor |ζ| × *c_max_* (KCl) have a higher goodness of fit than models utilizing the *c_max_* (KCl) and ζ × *c_max_* (KCl) descriptors. Regression coefficient values that failed Student’s *t*-test *p* > 0.5 are in bold in Table 5. It seems that the *k*‘ descriptor corresponding to the speed of silver NP oxidation is not significant in any of the linear models. This was the reason why the model No.5 was built without this descriptor. Nevertheless, this model is characterized by a sufficiently high value of the correlation coefficient *r* and can be used to predict the silver NP toxicity to yeast cells. Still, we cannot properly cross-validate this model due to the lack of experimental data. It should also be noted that the particle size *d*, proven in many studies to be a significant determinant of silver NP toxicity, appeared to be of low significance in QNAR models built in this study.

## 4. The Validity Testing of Using Various Descriptors to Predict the Toxicity of Silver NPs with Respect to Wide Array of Living Organisms

### 4.1. Danio Rerio Embryos

Our recent study [68] describes the impact of anionic, cationic, and amphoteric surfactants and cationic polymers on the physical and chemical properties, colloidal stability and behavior of silver nanomaterials, as well as on their toxicity. In particular, the LC_50_ values for zebrafish were also determined for some of the silver NP dispersions described in this article. Based on these data given in Table 6, it might be possible to evaluate the significance of correlation between the values of various descriptors, including the proposed new descriptor |ζ| × *c_max_* (KCl), and the toxicity of silver NP dispersions with respect to *Danio rerio* embryos. Table 7 shows the corresponding Pearson linear correlation coefficient (*r*) values.

Similarly to the toxicological data regarding *S. cerevisiae,* Table 7 shows that the correlation between the average particle size and the toxicity of the NPs is the lowest. The *k‘* descriptor, which reflects the rate of dissolution of silver NPs and generation of Ag^+^ ions, is characterized by the highest correlation coefficient values. The significance of the new proposed descriptor |ζ| × *c_max_* (KCl) was similar to that of the ζ-potential value which is a commonly used descriptor. The correlation coefficient value for the *S_sp_* descriptor was relatively high due to the fact that the Ag^+^ ions generation rate, which, in the case of *Danio rerio* embryos, is one of the most important factors and directly depends on the surface area of silver NPs.

### 4.2. Mycelial Fungi and Cyanobacteria

The influence of various descriptors on the toxicity of silver NPs with respect to other living microorganisms was evaluated in a similar manner. The obtained silver NP dispersion activity against eukaryotic phytopathogenic mycelial fungi *Rhizoctonia solani* J.G. Kühn and *Alternaria solani* Sorauer, as well as prokaryotic *Synechocystis* sp. PCC-6803 was studied. Effective concentration (EC_50_) of silver NPs which causes a two-fold decrease in the fungal mycelium growth is given in Table 8 together with the MIC values for *Synechocystis* sp. Table 9 presents the Pearson linear correlation coefficient (*r*) values for the correlation of various descriptors with the toxicity of silver NPs.

The EC_50_ and MIC values for capping agents in all cases exceeded the EC_50_ and MIC values for the corresponding dispersions of silver NPs. Therefore, the effect of the dispersions on *R. solani*, *A. solani* and *Synechocystis* sp. was mainly due to the action of silver NPs.

Toxicity of silver NPs has low correlation with parameters such as particle size or charge (see Table 9). As with zebrafish embryos, the *k‘* descriptor seems to be of greatest significance. At the same time, the influence of the new descriptor |ζ| × *c_max_* (KCl) turned out to be significantly larger than that of the traditional ζ-potential descriptor and the *c_max_* (KCl) descriptor reflecting the colloidal stability of NPs. Among all calculated parameters the descriptor |ζ| × *c_max_* (KCl) turned out to be the one that predicted the toxicity of silver NPs against *Synechocystis* sp. most efficiently. It should also be noted that in a solid malt agar medium, the influence of the colloidal stability of NPs on their activity is much larger compared with a liquid broth medium (e.g., one used to cultivate yeast or cyanobacteria) where the colloidal stability of silver NP dispersions tends to be higher.

## 5. Conclusions

After the quantitative evaluation of the variety of experimentally determinable structural, electrochemical and kinetic parameters of the dispersions, we have demonstrated that the particle electric charge (ζ-potential) should definitely be included in the relevant QNAR models for the evaluation of the silver NP toxicity, as well as at least one parameter demonstrating the colloidal stability of nanosilver dispersions. An example of the latter parameter is the maximum concentration of the coagulating agent that still allows the dispersion to keep its aggregate stability, or a new descriptor |ζ| × *c_max_* (KCl) suggested by us, which appears to be an integral parameter of the dispersion aggregate stability. At the same time, the traditional descriptors of a particle size or the studied potential descriptor characterizing the speed of silver ion generation in the oxidative dissolution of NPs have turned out to be less significant.

The validity testing of the proposed model for the theoretical prediction of silver NP toxicity using a wide range of living organisms has shown that this new descriptor correlates with toxicity much better compared to most traditionally used descriptors. Consequently, it seems promising in terms of being used not only in situations involving the rather narrow array of the objects tested, but also for the construction of silver NP toxicity models with respect to other living organisms.

## Figures and Tables

**Table 1:** The list of stabilizers of different chemical classes used for the synthesis of silver nanoparticles (NPs). Each aqueous dispersion contained 100 µg/mL of silver and the minimum amount of the capping agent *c (St)* needed to effectively stabilize silver NPs.

Structural Formula of a Stabilizer and Its Name	Abbreviation	*c (St)*, wt. %
Trisodium citrate 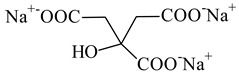	Citrate	0.045
Surfactants		
Sodium dodecyl sulfate 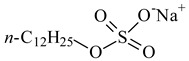	SDS	0.05
Sodium laureth sulfate, *m* = 1–3 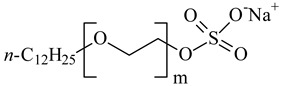	SLES	0.1
Sodium α-olefin sulfonate, *m* = 11–13 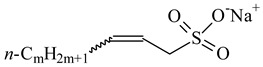	AOS	0.05
Coco trimethylammonium methoxy sulfate, *m* = 12–14 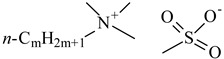	CTMAM	0.15
Alkoxy polyethylene glycol, *m* = 10–13 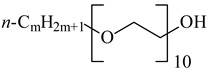	APG	0.5
Sodium coco aminodipropionate, *m* = 8–18 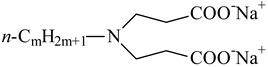	AMA	0.08
Sodium tallow amphopolycarboxyglycinate, *m* = 8–22, *p* = 2–3 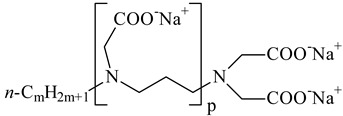	STAPCG	0.05
Nonylphenol ethoxylated 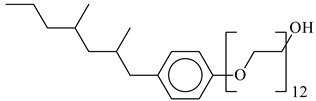	NPE	0.5
Polyoxyethylenesorbitan monooleate, *w* + *x* + *y* + *z* = 20 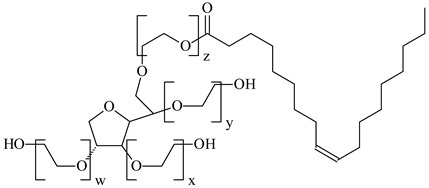	Tween-80	0.1
Didecyl dimethylammonium chloride 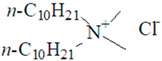	DDDMAC	0.02
Benzyldimethyl [3-(myristoylamino)-propyl] ammonium chloride 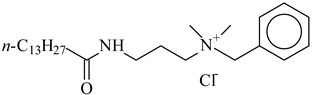	BDMMAC	0.02
Polymers		
Polyhexamethylene biguanide hydrochloride 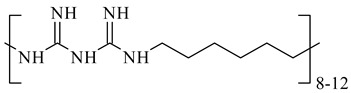	PHMB	0.01
Polyhexamethylene guanidine hydrochloride 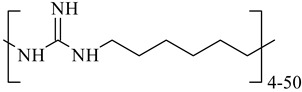	PHMG	0.01
Polydimethyldiallylammonium chloride 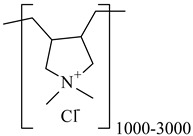	PDMDAAC	0.001
Polyacrylic acid 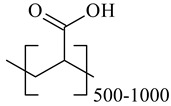	PAA	0.01
Ethoxylated polydimethylsiloxane 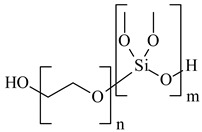	EPDMS	0.25
Xanthan gum, *n* = 5000–10,000 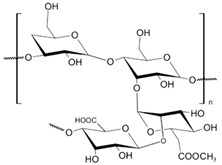	Xanthan gum	0.01

**Table 2:** Characteristics of the dispersions of silver NPs; *d*—mean diameter of silver NPs; *S*_sp_—specific surface; *c_max_* (KCl)—KCl concentration initiating the dispersion coagulation; *k*‘—averaged effective rate constant for oxidation of silver NPs by H_2_O_2_.

Capping Agent	ζ-Potential (mV)	*d* (Range of Variations) (nm)	*S*_sp_ (m^2^/g)	*c_max_* (KCl) (mM)	*k*‘ (L/mol·s) [55]
Citrate	−31 ± 1	7.5 (4–17)	48 ± 5	20 ± 5	0.11 ± 0.02
Polymers					
PAA	−43 ± 2	40 (10–50)	12 ± 1	180 ± 5	0.8 ± 0.1
Xanthan gum	−22 ± 2	10 (2–17)	41 ± 4	60 ± 5	0.2 ± 0.03
EPDMS	+8 ± 1	16 (9–30)	26 ± 3	120 ± 5	-
PDMDAAC	+31 ± 1	8.5 (6–17)	50 ± 5	80 ± 5	-
PHMG	+39 ± 2	7 (5–20)	52 ± 5	40 ± 5	0.9 ± 0.1
PHMB	+46 ± 1	3.5 (2–6)	82 ± 8	80 ± 5	1.2 ± 0.2
Surfactants					
AMA	−56 ± 1	9.5 (5–25)	43 ± 4	480 ± 5	0.9 ± 0.1
STAPCG	−54 ± 1	8.5 (6–20)	48 ± 5	1 130 ± 5	0.8 ± 0.1
AOS	−48 ± 2	8 (6–20)	42 ± 4	40 ± 5	0.9 ± 0,1
SLES	−46 ± 1	7.5 (4–17)	48 ± 5	30 ± 5	0.25 ± 0.04
SDS	−41 ± 1	10 (7–20)	38 ± 4	30 ± 5	0.6 ± 0.1
Tween-80	−18 ± 1	12 (9–25)	32 ± 4	300 ± 5	-
APG	−9 ± 1	11 (3–30)	35 ± 3	360 ± 5	1.8 ± 0.3
NPE	−12 ± 1	8 (5–20)	44 ± 5	410 ± 5	-
CTMAM	+34 ± 1	3 (2–6)	93 ± 6	170 ± 5	-
DDDMAC	+49 ± 1	10 (4–15)	38 ± 4	300 ± 5	1.4 ± 0.2
BDMMAC-B *	+54 ± 2	25 (12–40)	18 ± 3	240 ± 5	-
BDMMAC-G **	+23 ± 1	50 (25–100)	9 ± 3	110 ± 5	-

* silver NP dispersion obtained by the borohydride reduction method; ** silver NP dispersion obtained by the glucose reduction method.

**Table 3:** Minimum inhibitory concentration (MIC) of silver NPs capped with different stabilizers introduced into the nutrient-infused medium towards *S. cerevisiae* cells; the nanosilver/capping agent ratio is given in Table 1.

Capping Agent	MIC (µg/mL)	log_10_ (MIC)	Capping Agent	MIC (µg/mL)	log_10_ (MIC)
Citrate	50	1.699	Surfactants		
Polymers			AOS	12.5	1.097
PAA	12.5	1.097	SLES	50	1.699
Xanthan gum	25	1.398	SDS	25	1.398
EPDMS	50	1.699	Tween-80	25	1.398
PDMDAAC	25	1.398	APG	50	1.699
PHMG	6.25	0.796	NPE	25	1.398
PHMB	6.25	0.796	CTMAM	12.5	1.097
Surfactants			DDDMAC	3.12	0.495
AMA	6.25	0.796	BDMMAC-B *	3.12	0.495
STAPCG	6.25	0.796	BDMMAC-G **	12.5	1.097

* silver NP dispersion obtained by the borohydride reduction method; ** silver NP dispersion obtained by the glucose reduction method.

**Table 4:** Pearson linear correlation coefficients (*r*) between silver NP dispersion descriptors and their toxicity for yeast cells; |*r*| > 0.5 are in bold.

*r*	*d*	*S_sp_*	*c_max_* (KCl)	ζ	*k*‘	|ζ| × *c_max_* (KCl)	log_10_ (MIC)
*d*	-	**−0.76**	−0.07	0.05	0.01	−0.06	−0.11
*S_sp_*	-	-	−0.03	0.19	0.01	0.02	−0.06
*c_max_* (KCl)	-	-	-	−0.27	0.28	**0.93**	−0.32
ζ	-	-	-	-	0.48	−0.28	−0.40
*k*‘	-	-	-	-	-	0.12	−0.41
|ζ| × *c_max_* (KCl)	-	-	-	-	-	-	−0.48
log_10_ (MIC)	-	-	-	-	-	-	-

**Table 5:** Characteristics of QNAR linear models for the prediction of the toxicity of silver NP dispersions to yeast cells; parameter values that failed Student’s *t*-test (*p* > 0.5) are in bold.

Descriptor	Regression Coefficient	Model No.			
		1	2	3	4
-	*a*_0_	1.2 ± 0.3	1.2 ± 0.3	1.3± 0.2	1.4 ± 0.1
*d*	*a*_1_	**−0.01 ± 0.01**	**−0.01 ± 0.01**	−0.01 ± 0.01	−0.004 ± 0.006
*k*‘	*a*_2_	**0.06 ± 0.3**	**−0.05 ± 0.3**	**10^−4^ ± 0.2**	-
ζ	*a*_3_	−0.007 ± 0.004	−0.009 ± 0.004	−0.007 ± 0.003	−0.006 ± 0.002
*c_max_* (KCl)	*a*_4_	−0.7 ± 0.4	-	-	-
ζ × *c_max_* (KCl)	*a*_5_	-	0.014 ± 0.007	-	-
|ζ| × *c_max_* (KCl)	*a*_6_	-	-	−0.016 ± 0.006	−0.018 ± 0.005
**Statistics**					
Number of data points		12	12	12	19
*r*		0.69	0.71	0.78	0.74
Residual sum of squares		1.04	0.98	0.75	1.28

**Table 6:** LC_50_ values for silver NP dispersions in zebrafish embryos after 96 h of exposure.

Abbr. of NPs According to [68]	Capping Agent	LC_50_ (µg/mL)	R^2^	log_10_ (LC_50_)
Ag_I	SLES	0.219	0.993	−0.660
Ag_II	BDMMAC-B	0.956	0.998	−0.0195
Ag_III_cr_0.2	PHMB	2.917	0.999	0.465
Ag_IV_cr_12	AMA	0.515	0.999	−0.288
Ag_V	STAPCG	2.488	0.961	0.396

**Table 7:** Pearson linear correlation coefficient (*r*) values for silver NP dispersion descriptors and their toxicity for *Danio rerio* embryos log_10_ (LC_50_).

Descriptor	*d*	*S_sp_*	*c_max_* (KCl)	ζ	*k*‘	|ζ| × *c_max_* (KCl)
*r*	−0.13	0.41	0.43	0.42	0.8 *	0.42

* excluding the *k*’ value for silver NPs capped with BDMMAC-B, data are unavailable.

**Table 8:** Toxicity characteristics of silver NP dispersions with respect to *R. solani*, *A. solani* and *Synechocystis* sp.

Capping Agent	*R. Solani*		*A. Solani*		*Synechocystis* Sp.	
	EC_50_ (µg/mL)	log_10_ (EC_50_)	EC_50_ (µg/mL)	log_10_ (LC_50_)	MIC (µg/mL)	log_10_ (MIC)
APG	50	1.70	75	1.88	2	0.30
PHMB	10	1.00	25	1.34	-	-
AMA	10	1.00	25	1.34	0.5	−0.30
STAPCG	5	0.70	10	1.00	0.1	−1.00

**Table 9:** Pearson linear correlation coefficient (*r*) values for silver NP dispersion descriptors and their toxicity against *R. solani* and *A. solani* log_10_ (EC_50_) and *Synechocystis* sp. log_10_ (MIC).

Descriptor	*d*	*S_sp_*	*c_max_* (KCl)	ζ	*k*‘	|ζ| × *c_max_* (KCl)
*Rhizoctonia solani* J.G. Kühn						
*R*	0.46	−0.41	−0.52	0.28	0.96	0.74
*Alternaria solani* Sorauer						
*R*	0.35	−0.29	−0.67	0.36	0.93	0.84
*Synechocystis* sp. PCC-6803						
*R*	0.99	−0.98	−0.94	0.82	0.89	1.00

## References

[1] Nowack B., Krug H.F., Height M. (2011). 120 Years of Nanosilver History: Implications for Policy Makers. Environ. Sci. Technol..

[2] Deshmukh S.P., Patil S.M., Mullani S.B., Delekar S.D. (2019). Silver nanoparticles as an effective disinfectant: A review. Mater. Sci. Eng. C Mater. Biol. Appl..

[3] Santos A.C., Morais F., Simões A., Pereira I., Sequeira J.A.D., Pereira-Silva M., Veiga F., Ribeiro A. (2019). Nanotechnology for the development of new cosmetic formulations. Expert Opin. Drug Deliv..

[4] Bapat R.A., Chaubal T.V., Joshi C.P., Bapat P.R., Choudhury H., Pandey M., Gorain B., Kesharwani P. (2018). An overview of application of silver nanoparticles for biomaterials in dentistry. Mater. Sci. Eng. C Mater. Biol. Appl..

[5] Mathur P., Jha S., Ramteke S., Jain N.K. (2018). Pharmaceutical aspects of silver nanoparticles. Artif. Cells Nanomed. Biotechnol..

[6] Aziz Z., Abu S.F., Chong N.J. (2012). A systematic review of silver-containing dressings and topical silver agents (used with dressings) for burn wounds. Burns.

[7] Mehmood A. (2018). Brief overview of the application of silver nanoparticles to improve growth of crop plants. IET Nanobiotechnol..

[8] Lee S.H., Jun B.-H. (2019). Silver Nanoparticles: Synthesis and Application for Nanomedicine. Int. J. Mol. Sci..

[9] Gusev A.A., Kudrinsky A.A., Zakharova O.V., Klimov A.I., Zherebin P.M., Lisichkin G.V., Vasyukova I.A., Denisov A.N., Krutyakov Y.A. (2016). Versatile synthesis of PHMB-stabilized silver nanoparticles and their significant stimulating effect on fodder beet (*Beta vulgaris L.*). Mater. Sci. Eng. C Mater. Biol. Appl..

[10] Krutyakov Y.A., Kudrinsky A.A., Gusev A.A., Zakharova O.V., Klimov A.I., Yapryntsev A.D., Zherebin P.M., Shapoval O.A., Lisichkin G.V. (2017). Synthesis of positively charged hybrid PHMB-stabilized silver nanoparticles: The search for a new type of active substances used in plant protection products. Mater. Res. Express.

[11] De Souza T.A.J., Souza L.R.R., Franchi L.P. (2019). Silver nanoparticles: An integrated view of green synthesis methods, transformation in the environment, and toxicity. Ecotoxicol. Environ. Saf..

[12] Rezvani E., Rafferty A., McGuinness C., Kennedy J. (2019). Adverse effects of nanosilver on human health and the environment. Acta Biomater..

[13] Al-Sid-Cheikh M., Rouleau C., Bussolaro D., Oliveira Ribeiro C.A., Pelletier E. (2019). Tissue Distribution of Radiolabeled 110mAg Nanoparticles in Fish: Arctic Charr (Salvelinus alpinus). Environ. Sci. Technol..

[14] De Matteis V., Rinaldi R. (2018). Toxicity assessment in the nanoparticle era. Adv. Exp. Med. Biol..

[15] Do Amaral D.F., Guerra V., Motta A.G.C., de Melo e Silva D., Rocha T.L. (2019). Ecotoxicity of nanomaterials in amphibians: A critical review. Sci. Total Environ..

[16] Kalantzi I., Mylona K., Toncelli C., Bucheli T.D., Knauer K., Pergantis S.A., Pitta P., Tsiola A., Tsapakis M. (2019). Ecotoxicity of silver nanoparticles on plankton organisms: A review. J. Nanopart. Res..

[17] Yan A., Chen Z. (2019). Impacts of Silver Nanoparticles on Plants: A Focus on the Phytotoxicity and Underlying Mechanism. Int. J. Mol. Sci..

[18] Zakharova O., Gusev A., Skripnikova E., Skripnikova M., Krutyakov Y., Kudrinsky A., Mikhailov I., Senatova S., Chuprunov C., Kuznetsov D. (2015). Study of ecologo-biological reactions of common flax to finely dispersed metallurgical wastes. IOP Conf. Ser. Mater. Sci. Eng..

[19] Fourches D., Pu D., Tassa C., Weissleder R., Shaw S.Y., Mumper R.J., Tropsha A. (2010). Quantitative nanostructure-activity relationship modeling. ACS Nano.

[20] Kovalishyn V., Abramenko N., Kopernyk I., Charochkina L., Metelytsia L., Tetko I.V., Peijnenburg W., Kustov L. (2018). Modelling the toxicity of a large set of metal and metal oxide nanoparticles using the OCHEM platform. Food Chem. Toxicol..

[21] Palmer C. (2008). Animal Rights.

[22] Puzyn T., Rasulev B., Gajewicz A., Hu X., Dasari T.P., Michalkova A., Hwang H.-M., Toropov A., Leszczynska D., Leszczynski J. (2011). Using nano-QSAR to predict the cytotoxicity of metal oxide nanoparticles. Nat. Nanotechnol..

[23] Winkler D.A., Mombelli E., Pietroiusti A., Tran L., Worth A., Fadeel B., McCall M.J. (2013). Applying quantitative structure-activity relationship approaches to nanotoxicology: Current status and future potential. Toxicology.

[24] Mukherjee D., Royce S.G., Sarkar S., Thorley A., Schwander S., Ryan M.P., Porter A.E., Chung K.F., Tetley T.D., Zhang J. (2014). Modeling in vitro cellular responses to silver nanoparticles. J. Toxicol..

[25] Silva T., Pokhrel L.R., Dubey B., Tolaymat T.M., Maier K.J., Liu X. (2014). Particle size, surface charge and concentration dependent ecotoxicity of three organo-coated silver nanoparticles: Comparison between general linear model-predicted and observed toxicity. Sci. Total Environ..

[26] Luan F., Tang L., Zhang L., Zhang S., Monteagudo M.C., Cordeiro M.N.D.S. (2018). A further development of the QNAR model to predict the cellular uptake of nanoparticles by pancreatic cancer cells. Food Chem. Toxicol..

[27] Nomura T., Miyazaki J., Miyamoto A., Kuriyama Y., Tokumoto H., Konishi Y. (2013). Exposure of the Yeast Saccharomyces cerevisiae to Functionalized Polystyrene Latex Nanoparticles: Influence of Surface Charge on Toxicity. Environ. Sci. Technol..

[28] Akter M., Sikder M.T., Rahman M.M., Ullah A.A., Hossain K.F.B., Banik S., Hosokawa T., Saito T., Kurasaki M. (2017). A systematic review on silver nanoparticles-induced cytotoxicity: Physicochemical properties and perspectives. J. Adv. Res..

[29] Huynh K.A., Chen K.L. (2011). Aggregation kinetics of citrate and polyvinylpyrrolidone coated silver nanoparticles in monovalent and divalent electrolyte solutions. Environ. Sci. Technol..

[30] Beer C., Foldbjerg R., Hayashi Y., Sutherland D.S., Autrup H. (2012). Toxicity of silver nanoparticles—Nanoparticle or silver ion?. Toxicol. Lett..

[31] Kasemets K., Käosaar S., Vija H., Fascio U., Mantecca P. (2019). Toxicity of differently sized and charged silver nanoparticles to yeast *Saccharomyces cerevisiae* BY4741: A nano-biointeraction perspective. Nanotoxicology.

[32] Bhattacharya D., Samanta S., Mukherjee A., Santra C.R., Ghosh A.N., Niyogi S.K., Karmakar P. (2012). Antibacterial activities of polyethylene glycol, tween 80 and sodium dodecyl sulphate coated silver nanoparticles in bormal and multi-drug resistant bacteria. J. Nanosci. Nanotechnol..

[33] He D., Dorantes-Aranda J.J., Waite T.D. (2012). Silver nanoparticle—Algae interactions: Oxidative dissolution, reactive oxygen species generation and synergistic toxic effects. Environ. Sci. Technol..

[34] Lee B., Lee M.J., Yun S.J., Kim K., Choi I.H., Park S. (2019). Silver nanoparticles induce reactive oxygen species-mediated cell cycle delay and synergistic cytotoxicity with 3-bromopyruvate in *Candida albicans*, but not in *Saccharomyces cerevisiae*. Int. J. Nanomed..

[35] Käosaar S., Kahru A., Mantecca P., Kasemets K. (2016). Profiling of the toxicity mechanisms of coated and uncoated silver nanoparticles to yeast *Saccharomyces cerevisiae* BY4741 using a set of its 9 single-gene deletion mutants defective in oxidative stress response, cell wall or membrane integrity and endocytosis. Toxicol. Vitro.

[36] O’Doherty P.J., Khan A., Johnson A.J., Rogers P.J., Bailey T.D., Wu M.J. (2017). Proteomic response to linoleic acid hydroperoxide in *Saccharomyces cerevisiae*. FEMS Yeast Res..

[37] Niazi J.H., Sang B.I., Kim Y.S., Gu M.B. (2011). Global gene Response in *Saccharomyces cerevisiae* exposed to silver nanoparticles. Appl. Biochem. Biotechnol..

[38] Horstmann C., Campbell C., Kim D.S., Kim K. (2019). Transcriptome profile with 20 nm silver nanoparticles in yeast. FEMS Yeast Res..

[39] Babele P.K., Singh A.K., Srivastava A. (2019). Bio-Inspired Silver Nanoparticles Impose Metabolic and Epigenetic Toxicity to *Saccharomyces cerevisiae*. Front. Pharmacol..

[40] Gow N. (2004). Yeast Physiology. The Metabolism and Molecular Physiology of Saccharomyces Cerevisiae.

[41] Karathia H., Vilaprinyo E., Sorribas A., Alves R. (2011). *Saccharomyces cerevisiae* as a Model Organism: A Comparative Study. PLoS ONE.

[42] Vertelov G.K., Krutyakov Y.A., Efremenkova O.V., Olenin A.Y., Lisichkin G.V. (2008). A versatile synthesis of highly bactericidal Myramistin^®^ stabilized silver nanoparticles. Nanotechnology.

[43] Krutyakov Y., Klimov A., Violin B., Kuzmin V., Ryzhikh V., Gusev A., Zakharova O., Lisichkin G. (2016). Benzyldimethyl [3-(miristoylamino)-propyl] ammonium chloride stabilized silver nanoparticles (Argumistin^TM^) in medicine: Results of clinical trials for treatment of infectious diseases of dogs and perspectives for humans. Eur. J. Nanomed..

[44] Frisken B.J. (2001). Revisiting the method of cumulants for the analysis of dynamic light-scattering data. Appl. Opt..

[45] Vagabov V.M., Ivanov A.Y., Kulakovskaya T.V., Kulakovskaya E.V., Petrov V.V., Kulaev I.S. (2008). Efflux of potassium ions from cells and spheroplasts of *Saccharomyces cerevisiae* yeast treated with silver and copper ions. Biochemistry.

[46] Kutuzova I.A., Kokaeva L.Y., Pobendinskaya M.A., Krutyakov Y.A., Skolotneva E.S., Chudinova E.M., Elansky S.N. (2017). Resistance of helminthosporium solani strains to selected fungicides applied for tuber treatment. J. Plant Pathol..

[47] Rippka R., Deruelles J., Waterbury J.B., Herdman M., Stanier R.Y. (1979). Generic Assignments, Strain Histories and Properties of Pure Cultures of Cyanobacteria. J. Gen. Microbiol..

[48] Rakhimberdieva M.G., Kuzminov F.I., Elanskaya I.V., Karapetyan N.V. (2011). Synechocystis sp. PCC 6803 mutant lacking both photosystems exhibits strong carotenoid-induced quenching of phycobilisome fluorescence. FEBS Lett..

[49] Kudrinskiy A.A., Krutyakov Y.A., Olenin A.Y., Romanovskaya G.I., Vasilyeva S.Y., Lisichkin G.V. (2009). Sensitized fluorescence of silver nanoparticles in the presence of pyrene. J. Fluoresc..

[50] Krutyakov Y., Kudrinskiy A., Zherebin P., Yapryntsev A., Pobedinskaya M., Elansky S., Denisov A., Mikhaylov D., Lisichkin G. (2016). Tallow amphopolycarboxyglycinate-stabilized silver nanoparticles: New frontiers in development of plant protection products with a broad spectrum of action against phytopathogens. Mater. Res. Express.

[51] Krutyakov Y.A., Kudrinsky A.A., Olenin A.Y., Lisichkin G.V. (2010). Synthesis of highly stable silver colloids stabilized with water soluble sulfonated polyaniline. Appl. Surf. Sci..

[52] Henglein A. (1993). Physicochemical properties of small metal particles in solution: “Microelectrode” reactions, chemisorption, composite metal particles, and the atom-to-metal transition. J. Phys. Chem..

[53] Bae E., Park H.-J., Lee J., Kim Y., Yoon J., Park K., Choi K., Yi J. (2010). Bacterial cytotoxicity of the silver nanoparticle related to physicochemical metrics and agglomeration properties. Environ. Toxicol. Chem..

[54] Le Ouay B., Stellacci F. (2015). Antibacterial activity of silver nanoparticles: A surface science insight. Nano Today.

[55] Krutyakov Y.A., Kudrinskiy A.A., Zherebin P.M., Lisichkin G.V. (2019). Correlation between the rate of silver nanoparticle oxidation and their biological activity: The role of the capping agent. J. Nanopart. Res..

[56] Bae E.J., Park H.J., Park J.S., Yoon J.Y., Kim Y.H., Choi K.H., Yi J.H. (2011). Effect of Chemical Stabilizers in Silver Nanoparticle Suspensions on Nanotoxicity. Bull. Korean Chem. Soc..

[57] Abbaszadegan A., Ghahramani Y., Gholami A., Hemmateenejad B., Dorostkar S., Nabavizadeh M., Sharghi H. (2015). The Effect of Charge at the Surface of Silver Nanoparticles on Antimicrobial Activity against Gram-Positive and Gram-Negative Bacteria: A Preliminary Study. J. Nanomater..

[58] Carvalho P.M., Felício M.R., Santos N.C., Gonçalves S., Domingues M.M. (2018). Application of Light Scattering Techniques to Nanoparticle Characterization and Development. Front. Chem..

[59] Clogston J.D., Patri A.K. (2011). Zeta potential measurement. Methods Mol. Biol..

[60] Baalousha M., Afshinnia K., Guo L. (2018). Natural organic matter composition determines the molecular nature of silver nanomaterial-NOM corona. Environ. Sci. Nano.

[61] Afshinnia K., Marrone B., Baalousha M. (2018). Potential impact of natural organic ligands on the colloidal stability of silver nanoparticles. Sci. Total Environ..

[62] Römer I., White T.A., Baalousha M., Chipman K., Viant M.R., Lead J.R. (2011). Aggregation and dispersion of silver nanoparticles in exposure media for aquatic toxicity tests. J. Chormatorgr. A.

[63] Liu J., Hurt R.H. (2010). Ion release kinetics and particle persistence in aqueous nano-silver colloids. Environ. Sci. Technol..

[64] Andrews J.M. (2002). Determination of minimum inhibitory concentrations. J. Antimicrob. Chemother..

[65] Chernousova S., Epple M. (2013). Silver as antibacterial agent: Ion, nanoparticle, and metal. Angew. Chem. Int. Ed. Engl..

[66] Behra R., Sigg L., Clift M.J.D., Herzog F., Minghetti M., Johnston B., Petri-Fink A., Rothen-Rutishauser B. (2013). Bioavailability of silver nanoparticles and ions: From a chemical and biochemical perspective. J. R. Soc. Interface.

[67] Kudrinskiy A.A., Ivanov A.Y., Kulakovskaya E.V., Klimov A.I., Zherebin P.M., Khodarev D.V., Le A.-T., Le Tam T., Lisichkin G.V., Krutyakov Y.A. (2014). The Mode of Action of Silver and Silver Halides Nanoparticles against *Saccharomyces cerevisiae* Cells. J. Nanopart..

[68] Abramenko N., Demidova T.B., Krutyakov Y.A., Zherebin P.M., Krysanov E.Y., Kustov L.M., Peijnenburg W. (2019). The effect of capping agents on the toxicity of silver nanoparticles to *Danio rerio* embryos. Nanotoxicology.

